# Methanolic extract of Fennel (*Foeniculum vulgare*) escalates functional restoration following a compression injury to the sciatic nerve in a mouse model

**DOI:** 10.1002/fsn3.2033

**Published:** 2020-12-13

**Authors:** Javeria Maqbool, Haseeb Anwar, Javed Iqbal, Azhar Rasul, Ali Imran, Shoaib Ahmad Malik, Asghar Shabbir, Fazeela Ijaz, Faiqa Sajid, Rabia Akram, Tao Sun, Muhammad Imran, Ghulam Hussain, Saiful Islam

**Affiliations:** ^1^ Neurochemicalbiology and Genetics Laboratory (NGL) Department of Physiology Faculty of Life Sciences Government College University Faisalabad Pakistan; ^2^ Department of Neurology Allied Hospital Faisalabad Medical University Faisalabad Pakistan; ^3^ Department of Zoology Faculty of Life Sciences Government College University Faisalabad Pakistan; ^4^ Institute of Home and Food Sciences Government College University Faisalabad Pakistan; ^5^ Department of Biochemistry Sargodha Medical College University of Sargodha Sargodha Pakistan; ^6^ Department of Biosciences COMSATS Institute of Information Technology Islamabad Pakistan; ^7^ Center for Precision Medicine School of Medicine and School of Biomedical Sciences Huaqiao University Xiamen China; ^8^ University Institute of Diet and Nutritional Sciences Faculty of Allied Health Sciences The University of Lahore Lahore Pakistan; ^9^ Institute of Nutrition and Food Science University of Dhaka Dhaka Bangladesh

**Keywords:** *Foeniculum vulgare*, functional recovery, oxidative stress, peripheral nerve injury

## Abstract

Peripheral nerve injury (PNI) is one of the major health concerns faced by the community at present. Till now, available therapeutic approaches are ineffective to fully heal a nerve injury and to assure the functional recovery entirely. Natural compounds can prove attractive and effective drug candidates to bridge up this gap. In this scenario, the present study was designed to explore the role of methanolic extract of *Foeniculum vulgare* (*F. vulgare*) seeds in accelerating the function regain following a sciatic nerve injury in a mouse model. For this purpose, 12 adult healthy albino mice (BALB/C), 8–10 weeks old, were grouped as control (Ctrl, *n* = 6) and treatment (Trt, *n* = 6). The treated group was given methanolic extract of F. vulgare (200 mg/kg per day) started from the day of nerve crush until the end of the study. The sensorimotor function regain assessed by hot plate test, grip strength, and SFI assessments was found significantly (*p* < .05) ameliorated in the *F. vulgare‐*treated group. A prominent improvement in the muscle mass of the treated group further affirmed these effects. Furthermore, morphometric analysis of muscle fiber cross‐sectional area of tibialis anterior muscle between groups revealed a noticeable improvement in muscle fibers’ diameter of the treated group. Conclusively, these findings suggest that *F. vulgare* methanolic extract exhibits the potential to escalate functional recovery following a peripheral nerve injury. However, the real players of this extract and the mechanism involved in boosting functional restoration need to be dissected by further work.

## Graphical abstract

Neuroprotective effect of Methanolic extract of Fennel (*Foeniculum vulgare*)

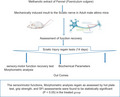

## 1 INTRODUCTION

The peripheral nervous system (PNS) comprises a complex network of delicate nerves that establish connections between the brain and spinal cord, the central nervous system (CNS), and the rest of the body for brain–body coordination. Nerves are delicate cord‐like structures that are probably at higher risk of getting injured by different kinds of traumatic injuries, developed as a result of roadside accidents, falls, or sharp lacerations. In the modern era, motor vehicle accidents are very common causing peripheral nerve injury (PNI) that eventually leads to severe and life‐changing disabilities (Miranda & Torres, 2016). Several rate‐limiting factors make the process of regeneration too slow to accomplish, yet an injured peripheral nerve can regenerate. As a result, the target muscle initiates atrophying, which further delays or even prohibits functional recovery (Houdek & Shin, 2015). A variety of therapeutic options are available, but they remain unsuccessful in attaining desired functional recovery at an optimal cost and accessibility. Ongoing neuroscience research continues to reveal new and alternative therapeutic options that could minimize or eliminate the disabilities of patients from physical dependency by promoting the healing mechanisms underlying such injuries (Hussain et al., 2020; Razzaq, Ahmad, et al., 2020). Plants bearing medicinal capability are abundantly available in the world and are famous for their manipulation as remedial agents to cure ailments for ages. They are highly enriched with several bioactive compounds having physiologically important biomedical activities (anti‐inflammatory, antioxidative, antimicrobial, antidiabetic, and analgesic) with minimal or no side effects (Hussain et al., 2018; Pant, Pant, Saru, Yadav, & Khanal, 2017). *Foeniculum vulgare* (belonging to *Apiaceae* family) is an aromatic plant with yellow flowers and feathery leaves, whose seeds are commonly used as a spice all over the world. It is one of the commonly used medicinal plants which also owns economic importance in addition to its beneficial medicinal effects (Badgujar et al., 2014). *Foeniculum vulgare* is highly enriched with vitamins and minerals including potassium, iron, thiamine, phosphorus, and vitamin C. The most important phytoconstituents of *Foeniculum vulgare* include phenolic acids, coumarin, tannins, flavonoids, and hydroxycinnamic acids (Kooti et al., 2015). The pharmacological activities of this plant are numerous such as antibiotic, antifungal, gastroprotective, antidiabetic, antioxidant, anti‐inflammatory, estrogenic, anticancer, hepatoprotective, antidepressant, hyperlipidemic, and antihypertensive. Having antioxidant, analgesic, and anti‐inflammatory properties, it can be assumed that fennel can be a good candidate to be considered as an alternative therapeutic agent in escalating functional recovery following nerve injuries. Recently, panch phoron, which is a blend of five spices including *Foeniculum vulgare,* has shown antinociceptive and anti‐inflammatory activities in sciatic nerve crush injury mice model (Gias et al., 2020). Our group has recently demonstrated the positive impact of crude fennel seed supplementation in a mouse model of PNI (Imran et al., 2019). Based on the abovementioned preliminary findings, the current study has been conducted to explore the potential role of methanolic extract of fennel seeds in functional reclamation in a mechanically induced sciatic nerve crush mouse model. The finding of this work will be helpful in the identification and characterization of the real player(s) that could be valuable drug candidates in the future.

## 2 MATERIALS AND METHODS

### 2.1 Animals

Healthy adult male Albino mice (BALB/C) were procured from and kept in the animal housing facility of the Department of Physiology, Government College University, Faisalabad. The mice chosen for this study were of weight around 25‐30g and age 8–10 weeks. They were kept in plastic rodent cages one mouse/cage, at room temperature (25 ± 2°C), ambient humidity of 41%–59%, and ad libitum supply of water and food. In the meantime, the instructions of the light and dark cycle of 12 hr were also maintained until the termination of the experiment. This study was authorized by the Institutional Animal Care and Ethics Committee with approval number 627.

### 2.2 Plant material processing, extraction, and study design

*F. vulgare* seeds were procured from the local market of Faisalabad, Pakistan, and were identified by the Department of Botany, Government College University, Faisalabad. The seeds were ground into a fine powder. Then, 500g powder was soaked into methanol with constant shaking for 7 days. It was assured that the methanol level must be 2–3 times more than the powdered material during the whole period of extraction. Afterward, the extract was filtered and the filtrate was rotary‐dried at 40–50°C under reduced pressure to solidify the extract.

All animals following acclimatization were divided equally into two groups. The control group (Ctrl, *n* = 6) was served with normal chow throughout the study period. The treatment group (Trt, *n* = 6) was served with *F. vulgare* methanolic extract containing diet since the day of nerve injury induction. The daily dose of *F. vulgare* methanolic extract was 200 mg/kg (Paul & Datta, 2017). The food and water intake along with average body weight was also measured throughout the experiment period.

### 2.3 Compression to Sciatic nerve

The sciatic nerve compression was induced in all mice following the acclimatization of five to six days by following the protocol mentioned in previous studies (Halter & Aguilar, 2010; Hussain et al., 2013; Imran et al., 2019; Junxiong et al., 2013). In brief, mice were anesthetized with a mixture of ketamine (70mg/kg) and xylazine (5mg/kg) by intraperitoneal injections. The skin was shaved smoothly at the midthigh region of the experimental hind paw. Following the sedation, the sciatic nerve was exposed and crushed precisely by utilizing a fine pair of forceps by applying a steady power for about 12–15 s. After making sure that the nerve was perfectly damaged by visual observation (a transparent ring would appear at the crushed site), the skin was sutured with 3–4 sutures and pyodine was applied at the surgery site to avoid infection. The hind paw with the sciatic nerve crush was taken as the ipsilateral paw, and the opposite hind paw was taken as the contralateral paw.

### 2.4 Sciatic functional index

The sciatic functional index (SFI) is an index to assess the pattern of rodents’ gait in experimental neurological studies. It was calculated by walking track analysis to figure out the regain in motor functions by following the procedure mentioned in previous studies. The hind paws were painted with ink, and the mouse was permitted to walk on a 50cm long and narrow wooden track with white paper on the floor. All the calculations from the most clearly inked prints of paws per run were measured and employed in the following formula to calculate SFI:

SFI = (‐ 38.3 x – EPL – NPL/ NPL) + (109.5 x ETS – NTS/ NTS) + (13.3 x EIT – NIT/ NIT) – 8.8

Here, EPL denotes experimental print length, which is the straight length from the top of the third toe to the heel. EIT is experimental intermediate toe, which is the distance between the second toe and the fourth toe. ETS is experimental toe spread, which is the distance from the first toe to the fifth toe. NPL stands for normal print length, which is from the top of the third toe to the heel. NIT (normal intermediate toe) is the distance between the second toe and the fourth toe. NTS (normal toe spread) is the distance from the first toe to the fifth toe. The experimental paw is the ipsilateral paw, and the normal paw is the contralateral paw (Aziz et al., 2019; Imran et al., 2019).

### 2.5 Muscle grip strength

The assessment of the griping force of hind limbs by grip strength meter is a reliable and optimal technique to assess the motor function regain following the sciatic nerve injury. It permits to calculate the in vivo strength of muscle by allowing the mouse to clamp a metal bar of grip strength meter (Bioseb, Chaville, France) which shows the gripping force in units of “N” on screen. Muscle grip strength was recorded individually for both hind paws ipsilateral and contralateral to injury site by using the method as mentioned in earlier studies (Imran et al., 2019; Rasul et al., 2019).

### 2.6 Hotplate test

The hotplate test is a commonly used behavioral test to analyze sensory function restoration following the sciatic nerve crush. This test allows measuring the sensory signal transmission in response to the in vitro thermal stimuli. Before performing the hotplate test, the mice were adapted to the nonfunctional hotplate instrument for 1–2 min (Aziz et al., 2019; Haas et al., 2010). Then, the mouse was positioned on the hot surface in such a way that the ipsilateral paw of the mouse remained touched with the surface. The temperature of the hotplate (SCILOGEX MS7‐H550‐S LED Digital 7x7 Hotplate Stirrer) was maintained as 56 ± 2°C. The mouse showed a response by either jerking or licking. The time taken by mouse to show response upon exposure to thermal stimuli was observed as paw withdrawal latency. For mouse, remained unresponsive until 30 s, the latency was recorded as 30 s and the thermal stimulus was abruptly removed to avoid injury. Similarly, the process was repeated two more times with an interval of 2 min in each reading and an average of three values was taken as the final reading (Aziz et al., 2019; Imran et al., 2019).

### 2.7 Biochemical parameters

At the end of the experiment, all mice were sacrificed after giving anesthesia. Their blood was collected and centrifuged to separate serum. The serum was then used for following biochemical analyses.

#### 2.7.1 Total oxidant status (TOS)

This test was performed to assess the oxidation state of the body. Excessive production of reactive oxygen species following an injury in the body is a common phenomenon that induces an imbalance between free radicals and the antioxidants. This creates oxidative stress which further exaggerates the pathological changes at the injury site. The level of oxidants in the serum samples was measured by adopting the method given by Erel, 2005. In this test, ferrous ion–dianisidine complex acts as a substrate. The oxidants present in the samples react with this complex to form a ferric ion. The ferric ion reacts with xylenol orange in an acidic medium to form a colored complex. The intensity of the color of this complex is directly proportional to the number of oxidants (responsible to convert the ferrous into ferric ion). The absorbance of this colored mixture was taken using an automated analyzer (Bio‐Lab 310) at 560nm wavelength. Hydrogen peroxide was used for the calibration of the analysis. The TOS concentration of samples was measured in μmol of H_2_O_2_ Eq./ L (Erel, 2005; Wu et al., 2017).

#### 2.7.2 Total antioxidant capacity (TAC)

Antioxidants are the defensive substances that protect the body from the harmful effects of free radicals (Rubio, 2016). These are the cell‐saving agents that compete for binding with the free radicals causing oxidative stress. This test was performed to evaluate TAC in the serum sample by following the method given by Erel, 2004, and has been adopted in earlier studies (Aziz et al., 2019; Erel, 2004). In this test, ABTS (2,2′‐azino‐di‐3‐ethylbenzothiazoline sulfonate) is used as a substrate. ABTS is allowed to be oxidized in the presence of hydrogen peroxide in the acidic medium. The oxidized ABTS solution is of green color. The addition of serum samples in this medium would tend to bleach depending upon the presence of antioxidants in it. The absorbance of this mixture was taken by an automated chemistry analyzer (Bio‐Lab 310) at the wavelength of 650nm. Vitamin C was used for calibration of the analysis. The units of TAC were expressed in mmol of Vit. C Eq./ L.

#### 2.7.3 Random blood glucose assessment

Keeping in view the influence of glucose levels in worsening the pathological events at the injury site, random glucose‐level assessment was done both before inducing the nerve injury and also 12 days postinjury in all mice by following the procedure published in earlier studies (Razzaq, Hussain, et al., 2020). For this, a blood drop was taken after pricking the mouse's tail on the glucometer (ACCU‐CHEK) strip, and glucose level was measured in units of mg/dl (appeared on glucometer monitor).

### 2.8 Muscle mass

The muscle mass was measured after dissecting them out from both hind limbs following the decapitation of mice to estimate the extent of muscle atrophy. The gastrocnemius and the tibialis anterior muscles of the hind limbs of both normal chow and fennel chow groups were used for mass evaluation (Aziz et al., 2019; Gargiulo et al., 2014; Razzaq, Ahmad, et al., 2020).

### 2.9 Morphometric analysis

After the dissection, tibialis muscles from both hind limbs were surgically taken out and were fixed in 10% neutral buffer formalin with pH 6.8 for 24–48 hr. Tissue was allowed for overnight washing to remove fixative and further dehydrated in a series of alcohol. Tissue was embedded in paraffin, and the section of 5µm was taken using a microtome. Sections were mounted on slides and stained with hematoxylin and eosin (H&E) stains. A compound microscope was used to visualize and evaluate the morphometric pattern of both contralateral and ipsilateral muscles at 40X. Finally, images were taken using a camera (Optika B1). Later on, the cross‐sectional area for individual muscle fiber appeared in an equally selected area of the image was measured using ImageJ, version 1.52. The mean of all fibers per image was measured and compared between groups (Pierce, Smith, Trojanowski, & McIntosh, 1998).

### 2.10 Statistical analysis

All data were expressed as mean ± *SEM*. GraphPad Prism, version 8.0, was used to perform data analyses. Two‐way ANOVA followed by Sidak's multiple comparisons test was used to compare the results. A value of *p* < 0. 05 was considered significant.

## 3 RESULTS

### 3.1 Effects of *F. vulgare* on body weight and food intake

**FIGURE 1:**
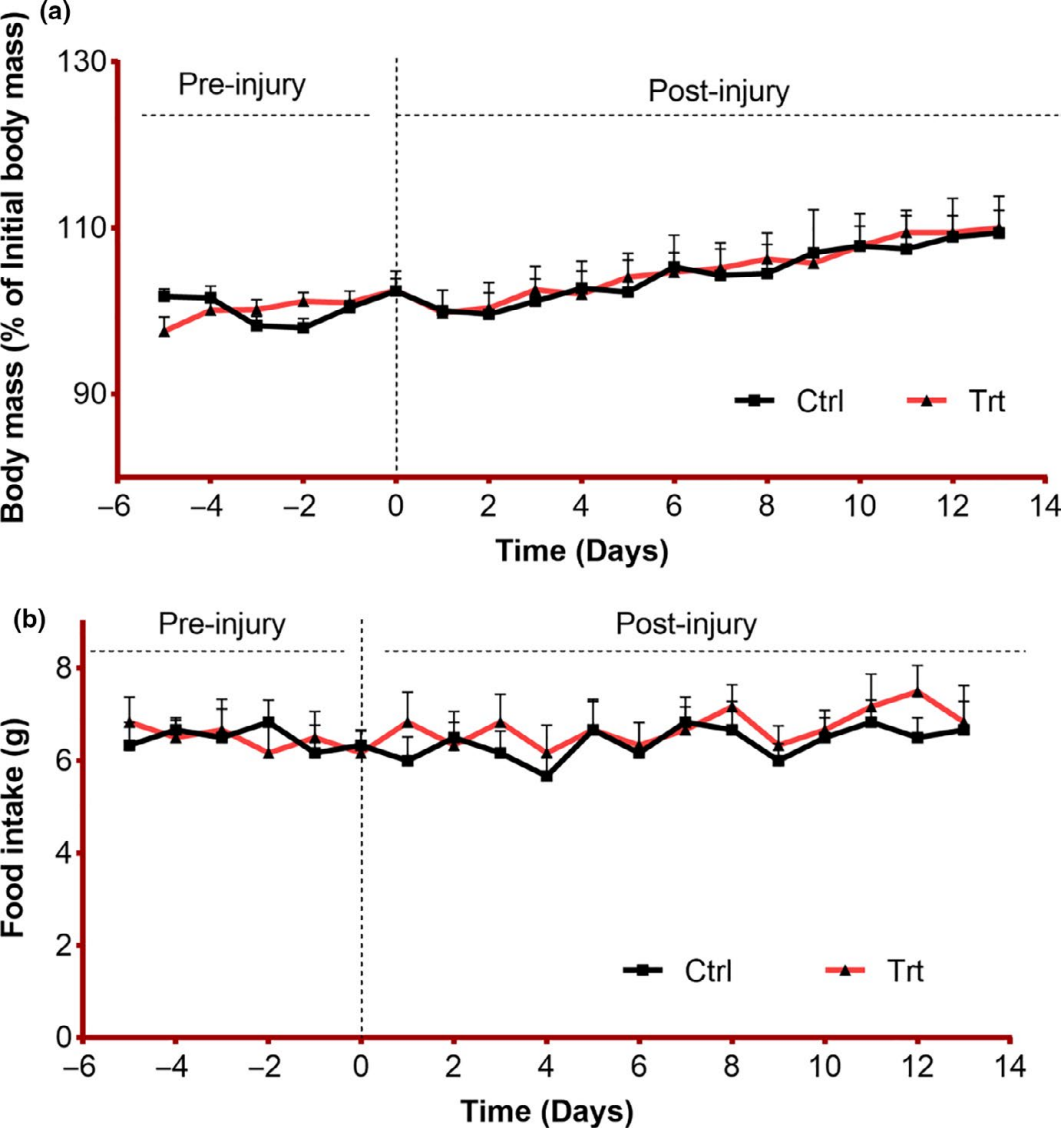
Influence of *F. vulgare* methanolic extract on body mass and food intake: Ctrl = control group fed on normal chow; Trt = treatment group fed on *F. vulgare* methanolic extract chow from the day of sciatic nerve injury induction to the end of the experiment: Results are represented as mean ± *SEM*: *n* = 6. (a) Body mass was measured daily during the whole experiment, two‐way repeated‐measures ANOVA represented the significant effect of time (*F*(18, 180)=7.28, *p* < .001), the nonsignificant effect of diet (*F*(1, 10)=0.01, *p* = .908), and a nonsignificant interaction between factors (time x diet) (*F*(18, 180)=7.28, *p* = .987). Sidak's multiple comparisons test predicted a nonsignificant effect of diet on body weight at all time points (*p* > .987). (b) Daily food intake was recorded for both groups. Two‐way repeated‐measures ANOVA represented a nonsignificant relation of time (*F*(18,180)=0.55, *p* = .93) and diet (*F*(1, 10)=1.44, *p* = .99) and a nonsignificant interaction between factors (*F*(18, 180)=0.27, *p* = .99). Sidak's multiple comparisons test predicted a nonsignificant effect of diet on daily diet consumption patterns in both groups (*p* > .99)

The body weight and food intake were measured in all groups daily right from the day of acclimatization to the end of the experiment. We found that neither the body mass nor food intake was affected by the addition of *F. vulgare* methanolic extract in the diet of mice. Also, the statistical analysis showed that there is a nonsignificant difference among all of the groups in body mass and food intake (*p* > .05) (Figure 1a,b).

### 3.2 Effect of *F. vulgare* on sensory‐motor function recovery

**FIGURE 2:**
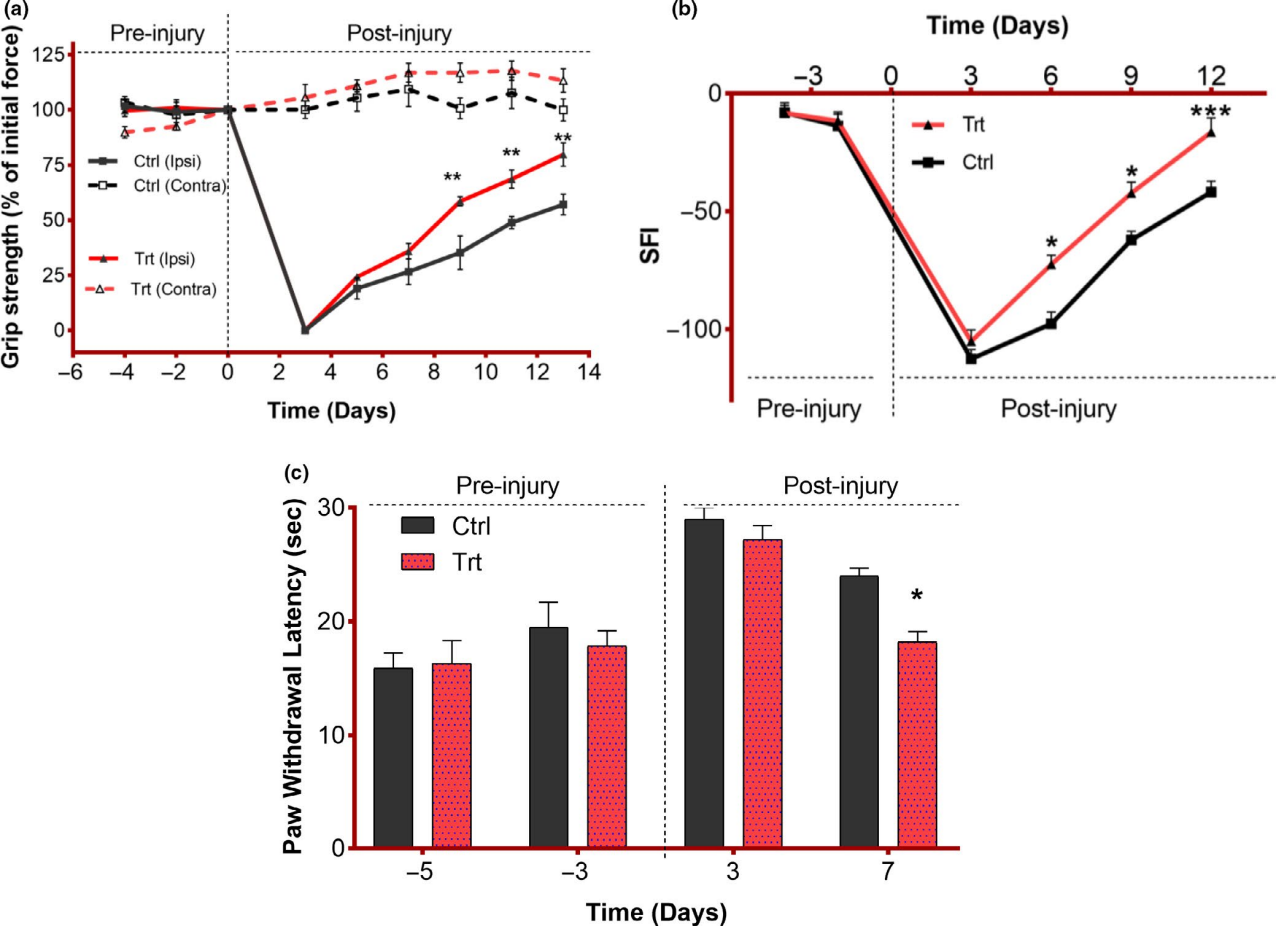
Influence of *F. vulgare* methanolic extract on sensorimotor function reclamation: Ctrl = control group fed on normal chow; Trt = treatment group fed on *F. vulgare* methanolic extract chow from the day of sciatic nerve injury induction to the end of the experiment: Results are mentioned as mean ± *SEM*: *n* = 6. (a) Measurement of muscle grip strength in mice for both hind paws (ipsilateral and contralateral to sciatic nerve crush) at different time points. Grip strength is expressed as a percentage of the average of initial forces recorded at days − 5 and − 3 pre‐injury per individual. Two‐way repeated‐measures ANOVA depicted a highly significant effect of time (*F*(8, 160)=9, *p* < .001) and diet (*F*(3, 20)=135, *p*=<0.001) and highly significant interaction between factors (*F*(24, 160)=79, *p* < .001). Sidak's multiple comparisons test revealed significant differences between both groups (for ipsilateral hind paws) at day 7 (**p = .001), day 9 (**p = .006), and day 11 (**p = .001) postinjury. (b) Measurement of the sciatic functional index (SFI) of mice at different time points. Two‐way repeated‐measures ANOVA depicted a significant impact of time (*F*(5, 50)=186, *p* < .001) and diet (*F*(1, 10)=17.2, *p* = .002) and a significant interaction between factors (*F*(5, 50)=3.78, *p* = .006). Sidak's multiple comparisons test represented significant differences between both groups at day 6 (*p = .001), day 9 (*p = .01), and day 11 (***p < .001) postinjury. (c) Measurement of paw withdrawal latency in mice on exposure to the thermal stimulus at different time points. Two‐way repeated‐measures ANOVA showed significant effects of time (*F* (3, 21)=31.3, *p* < .001) and diet (*F* (1, 7)=3.04, *p* = .125) and a nonsignificant interaction between factors (*F*(3, 21)=2, *p* = .14). Sidak's multiple comparisons test depicted a significant difference between both groups on day 7 (*p = .03) postinjury

The motor function regain following the injury was evaluated by using SFI and muscle grip strength. We found that the treated groups showed earlier motor function recovery (Figure 2a,b). The SFI and muscle grip strength were measured on different time points. The following graph depicts the same trend of motor function recovery as measured by SFI at day 6 (*p = .001), day 9 (*p = .01), and day 11 (***p < .001) of postinjury and muscle grip strength at day 6 (**p = .001), day 9 (**p = .006), and day 11 (**p = .001) of postinjury. The sensory function regain was measured by using the hotplate test on different days. Immediately following the nerve injury, the sensitivity capability was decreased in all groups. A significant decrease in paw withdrawal latency was observed on day 7 postinjury (*p* = .03) which pointed toward the quicker sensory function reclamation in the treated group which is assumed to be due to the potential of *F. vulgare* to promote axonal regeneration and ultimately quicker functional recovery (Figure 2c).

### 3.3 Effect of *F. vulgare* on oxidative stress and blood glucose

**FIGURE 3:**
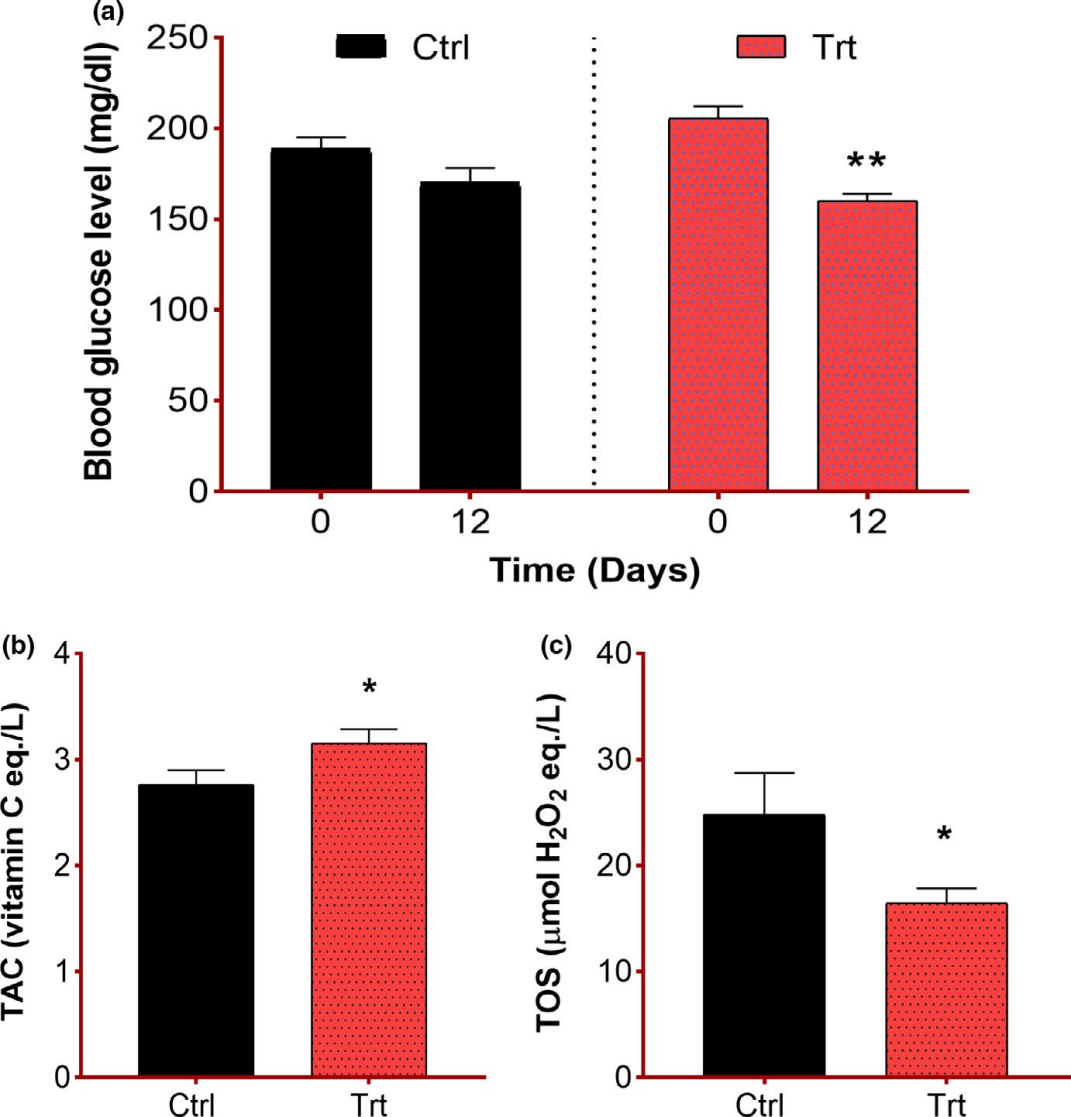
Influence of *F. vulgare* methanolic extract on systemic indices: Ctrl = control group fed on normal chow; Trt = treatment group fed on *F. vulgare* methanolic extract chow from the day of sciatic nerve injury induction to the end of the experiment: Results are manipulated as mean ± SEM. (a) Measurement of blood glucose level: Two‐way repeated‐measures ANOVA revealed a significant impact of time (F(1, 10)=19.3, *p* = .001), the nonsignificant effect of diet (F(1, 10)=0.43, *p* = .52), and a nonsignificant interaction of factors (F(1, 10)=3.35, *p* = .09). Sidak’s multiple comparisons test depicted a statistical difference in values (**p = .003) observed before and 12 days after injury induction in the treatment group. (b) Measurement of total antioxidant capacity in both groups: Unpaired t test (t=2.44, df=10) revealed a significant difference (*p = .01) between both groups. (c) Measurement of total oxidants status in both groups: Unpaired t test (t=2.01, *df*=10) depicted a significant difference (*p = .03) between both groups

The blood glucose level was recorded before inducing the injury and then 12 days postinjury. A significant reduction in glucose level was observed in the treatment group (**p = .003) in contrast to the control group where the pre‐ and postinjury difference in glucose level appeared to be nonsignificant (*p* > .05) (Figure 3a). These findings implicate that *F. vulgare* possesses hypoglycemic effects. The oxidative stress was measured in all groups at the end of the experiment. Oxidative stress measurement includes the evaluation of TOS and TAC levels. The TOS levels showed the extent of the presence of free radicals and TAC levels showed the antioxidant capacity in mice as affected by the *F. vulgare* methanolic extract diet. A significant increase in TAC values (*p = .03) and a decrease in TOS (*p = .03) had been shown by the treatment group which confirms the antioxidative capability of this plant.

### 3.4 Effects of *F. vulgare* on muscle mass

**FIGURE 4:**
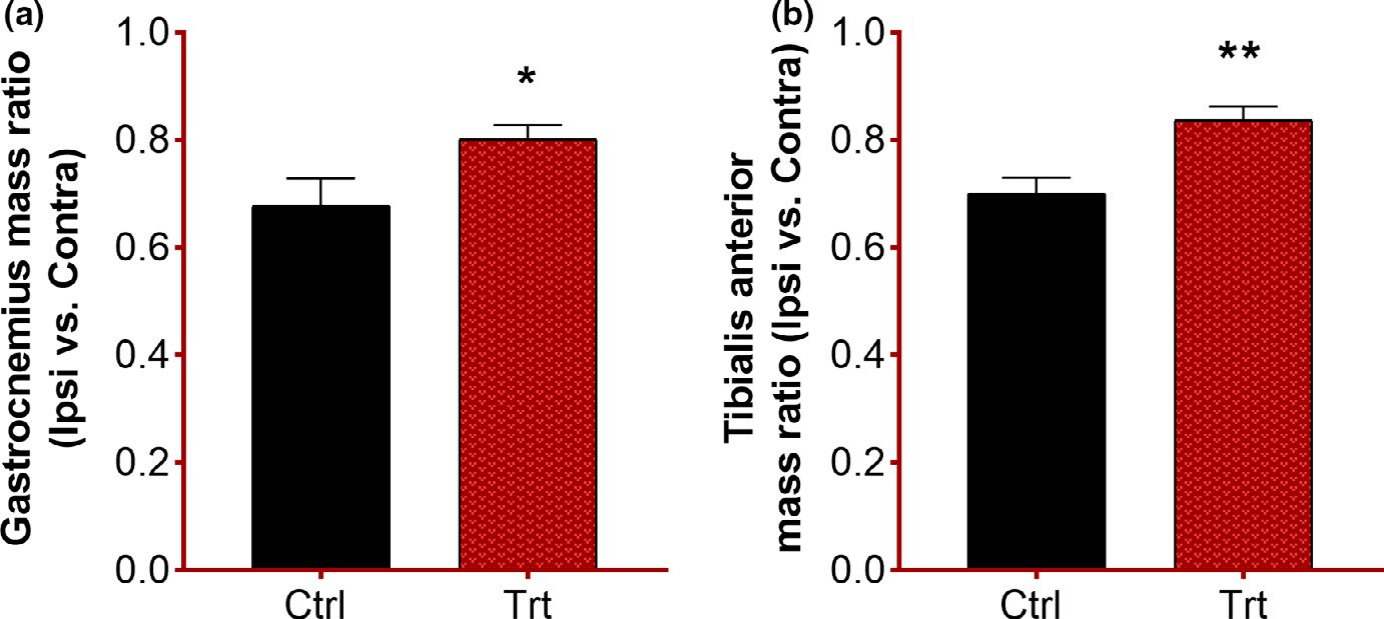
Influence of *F. vulgare* methanolic extract on skeletal muscle mass restoration: Ctrl = control group fed on normal chow; Trt = treatment group fed on *F. vulgare* methanolic extract chow from the day of sciatic nerve injury induction to the end of the experiment: Results are given as mean ± *SEM*. (a) Gastrocnemius muscle mass ratio; unpaired *t* test (t = 2.20, *df* = 10) showed significant difference between groups (*p = .02). (b) The tibialis anterior mass ratio; unpaired *t* test (t = 3.57, *df* = 10) showed a significant difference between groups (**p = .002)

The muscle mass was measured to access the extent of muscle atrophy as a result of the poor transfer of stimulus to the target muscle. A statistically significant increase in gastrocnemius (*p = .02), as well as tibialis anterior mass (**p = .002) in the treated group, was observed. This indicates the recovery process as increased mass is a sign of muscle re‐innervation in the treated group (Figure 4a,b).

### 3.5 Effect of *F. vulgare* on muscle fibers’ morphology

**FIGURE 5:**
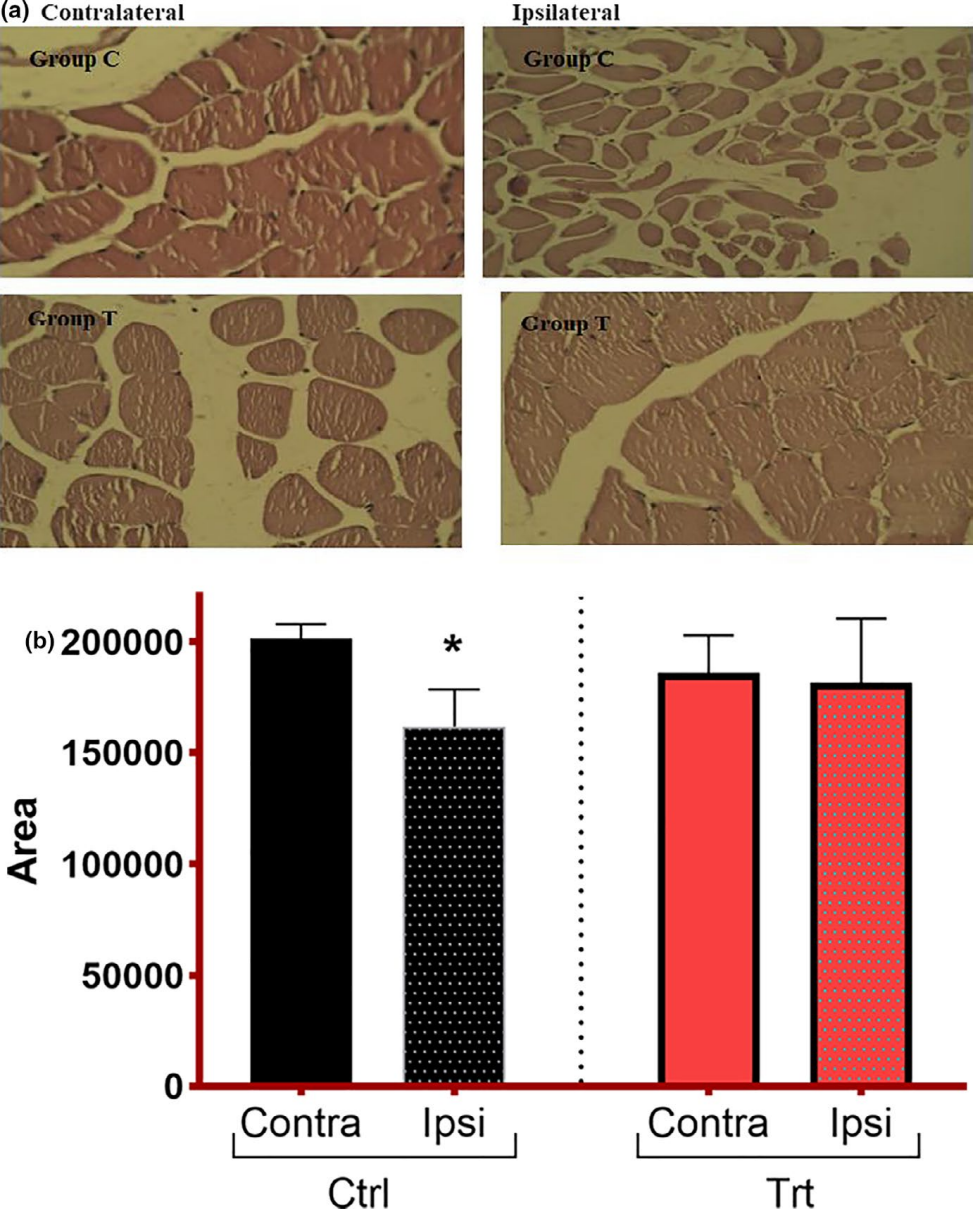
(a) Cross section of tibialis anterior muscle viewed under a compound microscope; H&E‐stained images taken at 40×. Group C = control; group T = treatment. (b) Influence of *F. vulgare* methanolic extract on tibialis muscle fiber's cross‐sectional area: Ctrl = control group fed on normal chow; Trt = treatment group fed on *F. vulgare* methanolic extract chow from the day of sciatic nerve injury induction to the end of the experiment: Results are expressed in mean ± *SEM*. Unpaired *t* test (t = 2.19, *df* = 10) showed significant difference (*p = .02) between ipsi and contra muscles in control group while nonsignificant difference ((*p* = .45), t = 0.12, *df* = 10) between ipsi and contra muscles in the treatment group

An injury to nerve results in an interruption of electrical signals to the target muscle. Muscle fibers undergo atrophy in the prolonged absence of such signals. A small fiber cross‐sectional area and irregular morphology are generally used to assess the health status of muscle fibers. The comparison of the cross‐sectional area of ipsilateral and contralateral muscles in the control group shows the presence of dystrophy (Figure 5). However, the treatment group exhibits a statistically nonsignificant difference (*p* = .45), of muscle's cross‐sectional areas that affirms the ameliorated restoration of muscle fiber health in response to treatment. It depicts a prominent impact of *F. vulgare* methanolic extract in speeding up the axonal regeneration (Figure 6).

## 4 DISCUSSION

*F. vulgare* is renowned for its medicinal properties, and its use as spice and folk medicine is well‐acknowledged. Although there are plenty of data on its antioxidant, antidepressant, neuroprotectant, and anxiolytic effects, yet its role in improving functional recovery following PNI has never been dissected. Recent studies have reported the potential neuroprotective effects (Gias et al., 2020) and neuroregenerative effects depicting functional recovery (Imran et al., 2019) following sciatic nerve crush injury in a mouse model of fennel seeds. So, here we investigate the possible effect of *F. vulgare* methanolic extract on escalating functional recovery.

Our findings indicate that the addition of this plant in the diet of animals does not alter the food preference of animals suggesting that the selected dose is safe and any improvement would be due to this plant extract. An accelerated sensory‐motor function restoration (evaluated by hot plate test, grip strength measurement, and SFI) was observed in the treated group. The current study reveals the significantly accelerated grip force in the treated group (>60% at day 9 and > 70% on day 11 and > 80% at day 13 postinjury). This shows that the trend of ameliorated grip force in the treatment group of the current study is almost similar to that found in an earlier study done by Imran et al., 2019, and Aziz et al., 2019, who used the crude powder of fennel seed and *Cannabis sativa,* respectively.

Hind limb skeletal muscles (tibialis anterior and gastrocnemius) are directly innervated by the sciatic nerve, and these muscles are compromised upon sciatic nerve compression. As, it is well evident that sustained loss of nerve conduction to muscle leads to muscular shrinkage which causes further functional loss due to injury (McKinnell & Rudnicki, 2004; Sandri, 2008). If this situation persists for long, it can cause permanent muscular dystrophy which would be irreversible to recover (Hussain et al., 2020). Thus, in time neuroregeneration accelerating therapeutic moiety is required to avoid permanent muscular damage. Moreover, muscle atrophy further delays the regeneration of damaged nerve via reducing the production of neurotrophic growth factors (Tuffaha et al., 2016). Mass of both gastrocnemius and tibialis anterior was restored in the treated group suggest. Our findings are in accordance with the already reported observations made by Imran et al., 2019.

Histopathological comparison of tibialis anterior muscle from ipsilateral and contralateral sides in each group clears/supports the findings as observed by behavioral analyses. Each muscle fiber was completely evaluated, and a cross‐sectional area was measured by ImageJ. The comparison of the area of muscle fibers’ cross section between ipsilateral and contralateral muscles in the treated group was found to be nonsignificant in contrast to the control group which showed significant differences in ipsilateral and contralateral muscle fiber's cross section. It reveals that affected muscle may start receiving almost normal conduction impulses from the nerve. *F. vulgare* treatment reduces muscle dystrophy and retained normal shape and muscle area. These findings highly suggest that *F. vulgare* methanolic extract would accelerate the nerve regeneration and restore the neuromuscular conduction; therefore, the ipsilateral muscle appeared to be healthy like the contralateral one.

The glucose‐lowering effect of fennel seeds is well evident from the previous literature (Godavari et al., 2018), and here, we also noted a significant decrease in the blood glucose level in the treated group. Oxidative stress is one of those major pathological phenomena which occur and exacerbate the pathological events at the injury site (Razzaq, Hussain, et al., 2020). Free radicals’ generation at the nerve injury site worsens the tissue damage and delay the neuroregenerative mechanisms. However, studies have highlighted that *F. vulgare* represents free radical scavenging and strong antioxidative properties (Imran et al., 2019; Oktay et al., 2003). Furthermore, the anti‐inflammatory and analgesic activities of *F. vulgare* have also been explored (Choi & Hwang, 2004). In the current study, comparatively improved TAC value and reduced TOS level in *F. vulgare* extract‐treated groups provide evidence about the oxidative stress combating properties of *F. vulgare* extract. Moreover, the results of this study strongly support our previously published findings that it restores the functional reclamation after nerve injury.

## 5 CONCLUSION

The findings of the current study suggest that the methanolic extract of *F. vulgare* accelerates the restoration of neuronal function following a mechanically induced injury to the sciatic nerve. Based on current observations, it can be suggested that these ameliorating effects can be accredited due to the antioxidative capability of the extract, but this requires further and detailed investigations. Further studies are required to figure out all possible mechanisms behind this improved functional recovery, and to explore the bioactive compound/compounds and their characterization that is/are potent to accelerate functional recovery following PNI. It will pave the way to develop cost‐effective therapeutic products.

## CONFLICT OF INTEREST

No conflict of interest stated from authors.

## ETHICAL APPROVAL

The study design and use of the animal model (mouse) for the current project were approved by the Institutional Review Board (IRB), Government College University, Faisalabad, Pakistan, having approval number 627.
